# Ruptured Ectopic Pregnancy, Ovarian Torsion, Dermoid Cyst, Leiomyomata, and Endometriosis: A Case Report of a Pelvic Quintet

**DOI:** 10.7759/cureus.66884

**Published:** 2024-08-14

**Authors:** Arianne Shipp, Wanda I Torres

**Affiliations:** 1 Osteopathic Medicine, Nova Southeastern University Dr. Kiran C. Patel College of Osteopathic Medicine, Clearwater, USA; 2 Obstetrics and Gynecology, Nova Southeastern University Dr. Kiran C. Patel College of Osteopathic Medicine, Clearwater, USA; 3 Obstetrics and Gynecology, Suncoast Women's Care, Trinity, USA

**Keywords:** dermoid cyst, ectopic pregnancy, ovarian torsion, uterine leiomyomata, endometriosis, ruptured ectopic pregnancy

## Abstract

The chances of a female of reproductive age presenting with a ruptured ectopic pregnancy are relatively low. Ectopic pregnancies make up 1-2% of all pregnancies and 20% of ectopic ruptures. The chances of a patient with an ovarian torsion with a dermoid cyst are also low. The incidence of ovarian torsions is 2-5%, and a dermoid cyst is found in 25% of all ovarian torsions. The odds of a single patient presenting with both a ruptured ectopic pregnancy and ovarian torsion with a dermoid cyst, along with other pathologies, including fibroids and endometriosis, are exceptionally improbable but not impossible. We present a case of a 32-year-old gravida 1 para 0000 (G1P0) female who presented to the emergency department (ED) after five weeks of amenorrhea with light vaginal bleeding and severe left lower quadrant abdominal pain. A transvaginal ultrasound (TVUS) was performed and was questionable but unclear for an ectopic pregnancy. A diagnostic laparoscopy was indicated and confirmed the diagnoses of a left ruptured ectopic pregnancy with hemoperitoneum, right ovarian torsion with a right ovarian dermoid cyst, multiple subserosal leiomyomas, and endometriosis of the posterior cul-de-sac. Given the unclear TVUS results, the ultimate decision to perform a diagnostic laparoscopy was largely based on the patient’s history and presenting symptoms. This case demonstrates a pelvic quintet, five rare pelvic anomalies, in a single patient who received a potentially lifesaving salpingectomy, right cystectomy, and right ovarian detorsion.

## Introduction

Female patients of reproductive age who present with pelvic pain and vaginal bleeding can have a wide array of pathologies. Depending on gestational status and gynecological history, these can range from chronic pain with menses to acute pain due to pregnancy complications. Initial evaluation of acute pelvic pain and vaginal bleeding in early pregnancy is with a speculum examination, transvaginal ultrasound (TVUS), and β-subunit of human chorionic gonadotropin (B-hCG) [1]. Other diagnostic testing may include urinary analysis, complete blood count, sexually transmitted disease testing, computerized tomography (CT), and hormone assay. If imaging is questionable for ectopic pregnancy, then a dilatation and curettage (D&C) and/or laparoscopy may be useful, as seen in this particular case [2].

Here, we present a pregnant patient with five weeks of amenorrhea who developed severe left lower quadrant abdominal pain with light vaginal bleeding approximately five days after testing positive with an at-home pregnancy test. Following a questionable TVUS, a diagnostic laparoscopy was indicated for concern of an ectopic pregnancy. During the pelvic exam and laparoscopy, the following diagnoses were confirmed: left ruptured ectopic pregnancy with hemoperitoneum, right ovarian torsion with right ovarian dermoid cyst, multiple leiomyomas, and endometriosis of the posterior cul-de-sac. This resulted in a left salpingectomy, detorsion of the right ovary, and cystectomy of the right ovarian dermoid cyst.

## Case presentation

A 32-year-old gravida 1 para 0000 (G1P0) female patient presented to the emergency department (ED) in June 2023 with severe left lower quadrant abdominal pain, light vaginal bleeding, and dysuria. She reported a positive home pregnancy test five days prior to evaluation. Her last known menstrual period was five weeks prior to this evaluation. She denied any current medication use but endorsed a history of progesterone-only oral contraceptive use. Her last use of birth control was unknown.

Her medical history included mitral valve prolapse, migraines with aura, acne, unspecified irregular periods since 2021, mildly elevated bilirubin found on routine blood work, and mildly elevated white blood cell (WBC) count found on routine blood work. Her mitral valve prolapse was managed with prophylactic antibiotics for dental work. The patient denied any allergies to food or medications. There was no history of sexually transmitted diseases, as reported by the patient. The patient’s family history consisted of a maternal grandmother with breast cancer and attenuated familial adenomatous polyposis. There was no history of alcohol, tobacco, or other substance abuse at the time of evaluation.

The patient was married, sexually active, and started planning for pregnancy in 2022, per medical records. Her last pap smear and gynecological exam in 2021 were negative. However, in 2021, her prolactin was mildly elevated at 30.2 ng/mL, and a pelvic ultrasound (US) indicated for irregular menstruation showed evidence of a left-sided hydrosalpinx. In 2022, a hysterosalpingogram was performed, and the report showed normal anatomy with patent fallopian tubes bilaterally.

**Figure 1:**
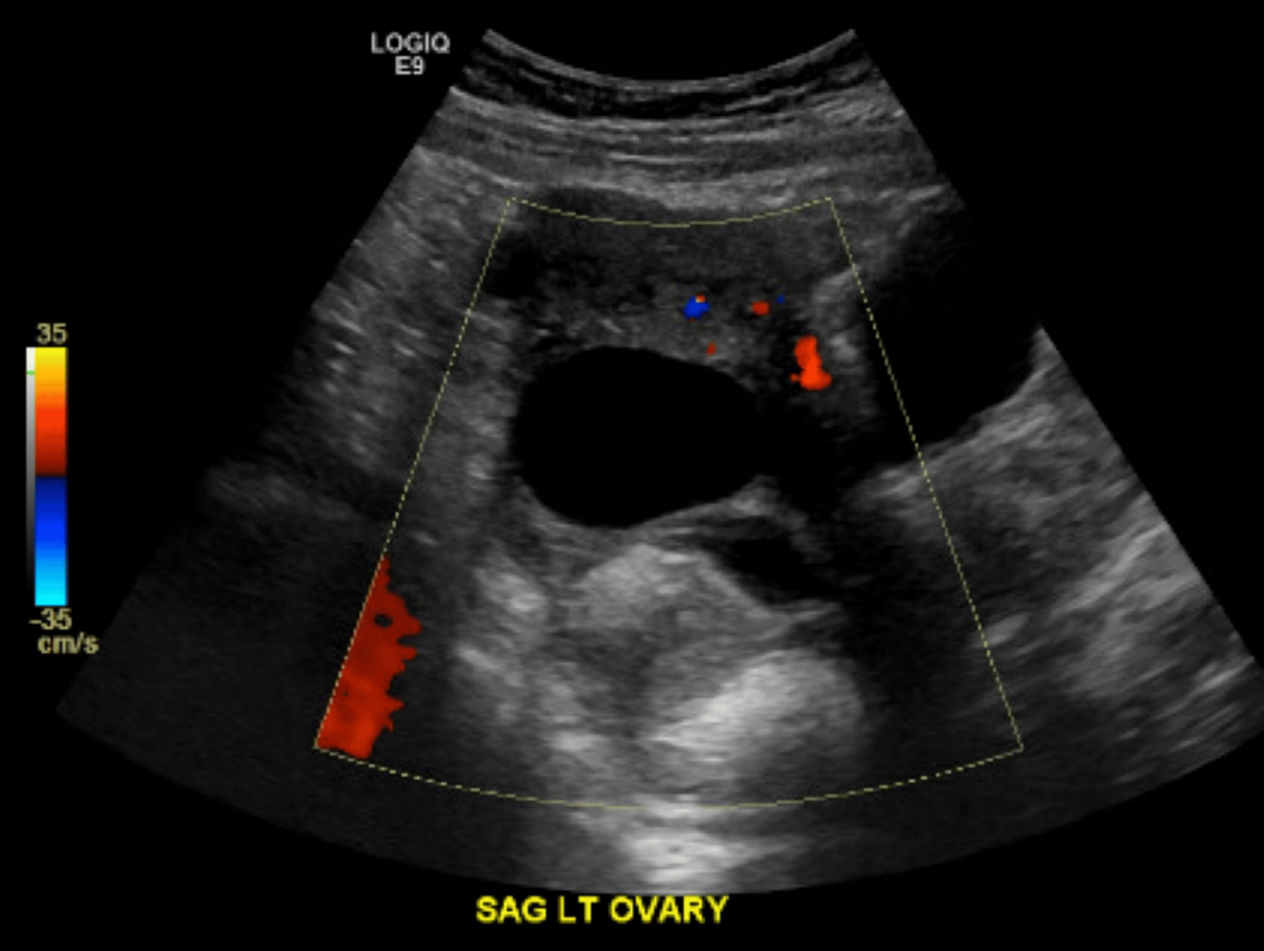
Ultrasound sagittal view of the left ovary This ultrasound report shows a sagittal view of the patient's left ovary measuring 9.3 × 5.5 × 9.8 cm, which is significantly larger than the right ovary, measuring 2.6 × 1.9 × 1.9 cm. There is a 4.2 cm cystic focus in the left ovary and a 7.7 cm left ovarian lesion, which was stated with uncertainty in the radiology report as a possible endometrioma.

Upon evaluation in the ED, the patient was in moderate distress with soft, non-distended left lower quadrant and left flank tenderness. Due to the severity of the patient’s pain and acute abdomen, the gynecological exam was to be deferred until imaging was performed or until under anesthesia. Vitals were in the normal range, including a BMI of 24.8 kg/m^2^. Her B-hCG was 2,317.20 mIU/mL, which was consistent with a five-week pregnancy [3]. Her blood type was B+ with a negative antibody screen. The WBC was slightly elevated at 11,100/mm^3^, and the monocytes were at 10.8%. A TVUS was indicated for early pregnancy bleeding and cramping. The report from the TVUS was as follows: a tiny cystic focus in the fundal endometrium that may represent an early intrauterine gestational sac; pseudogestational sac and spontaneous abortion in progress or occult ectopic pregnancy cannot be excluded; right ovary measures 2.6 × 1.9 × 1.9 cm with probable corpus luteum in the right ovary; left ovary measures 9.3 × 5.5 × 9.8 cm with 4.2 cm cystic focus in left ovary and a 7.7 cm left ovarian lesion suspicious for endometrioma; and mild free fluid in the pelvis (Figure 1-Figure 5).

**Figure 2:**
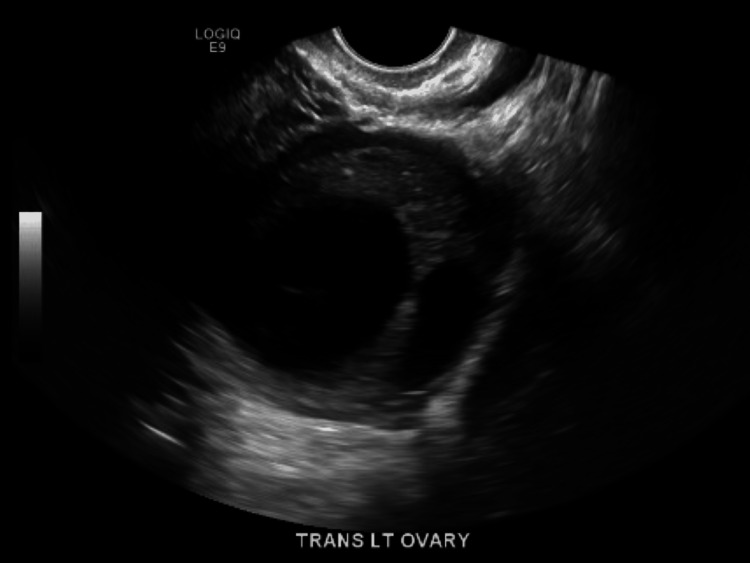
Ultrasound transvaginal view of the left ovary This is another view of the left ovary, showing the size of the left ovary, cystic focus, and left ovarian lesion.

**Figure 3:**
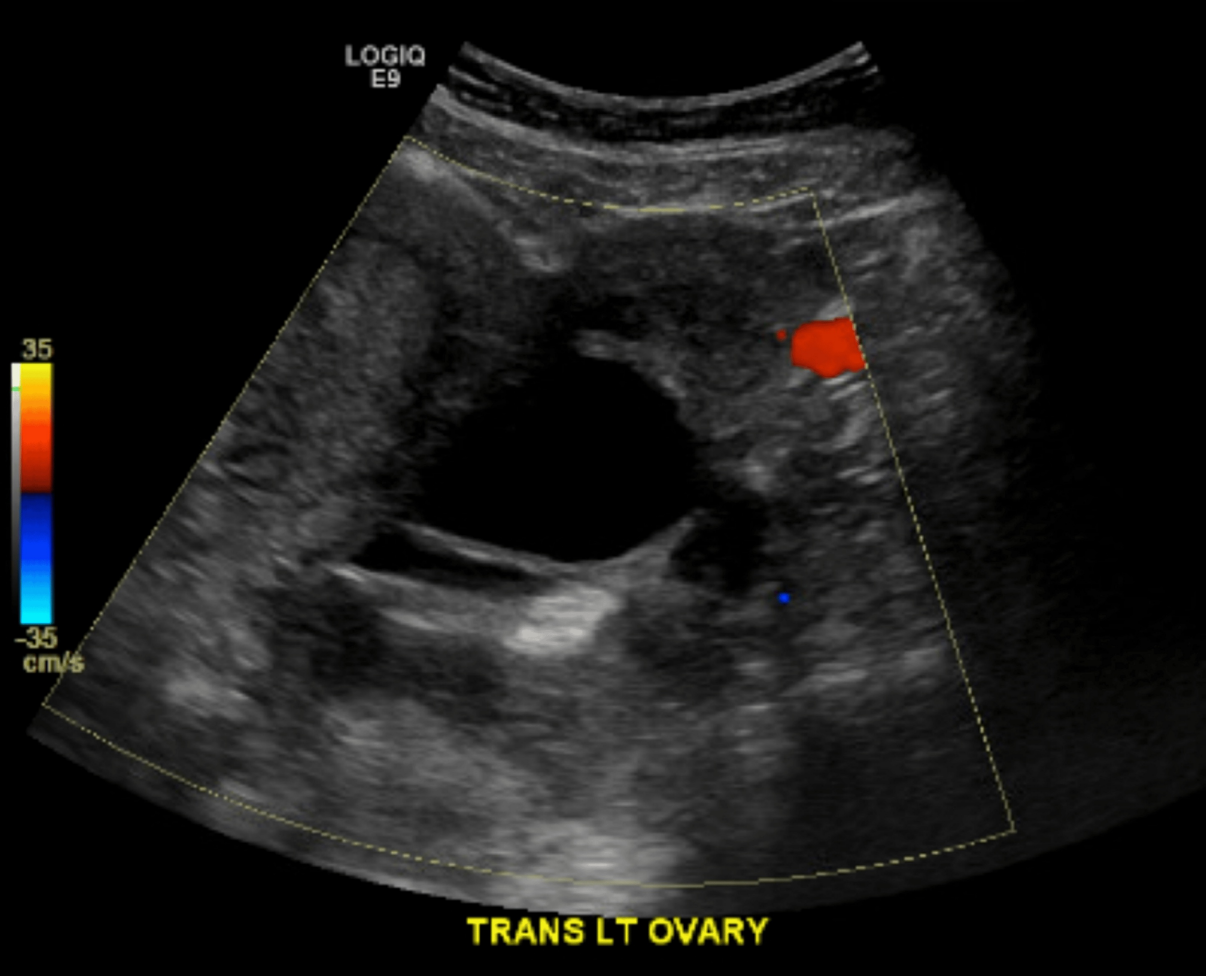
Ultrasound transvaginal view of the left ovary This is another transvaginal view of the left ovary showing the size, cystic focus, and lesion.

**Figure 4:**
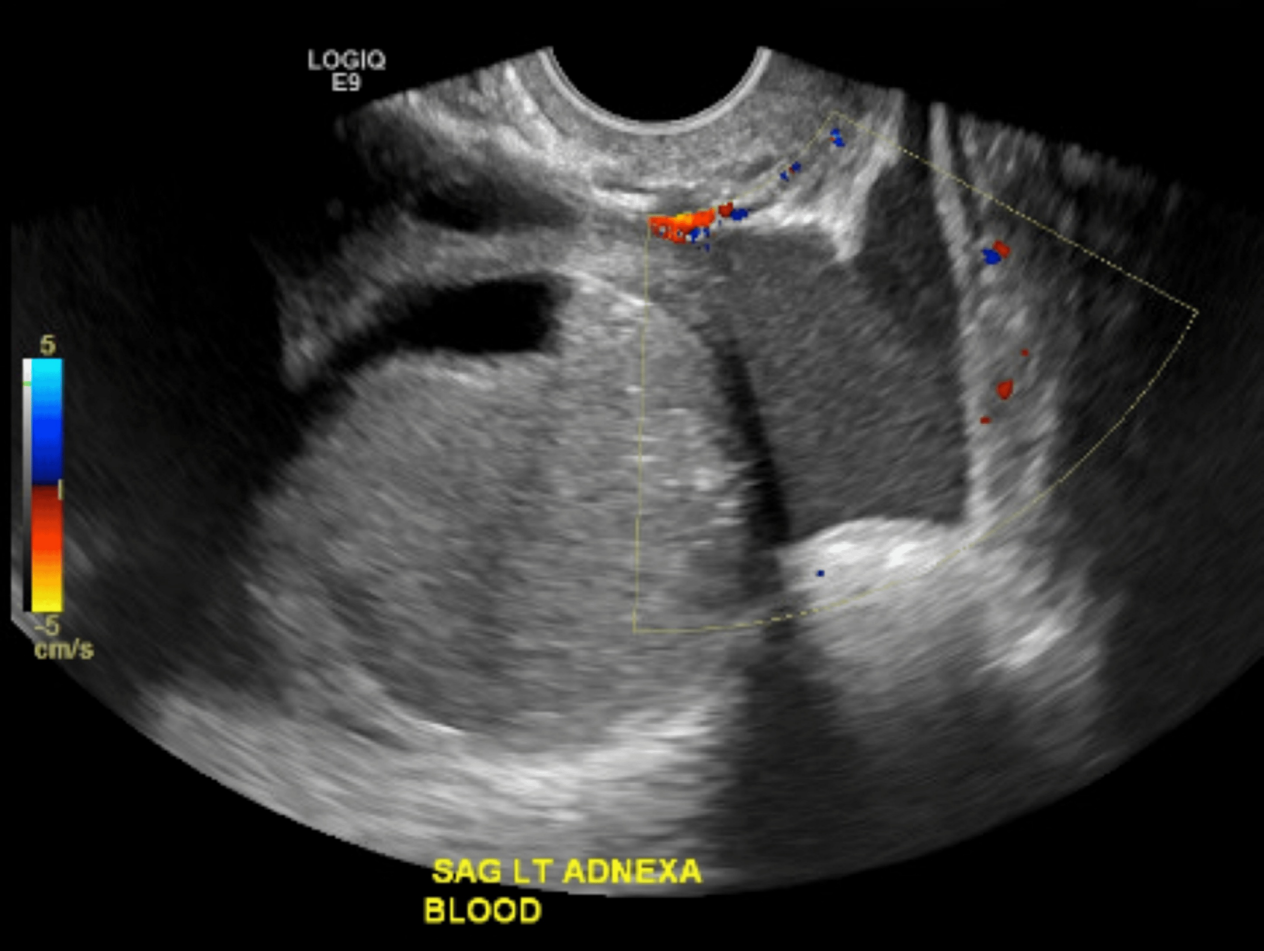
Ultrasound sagittal view of the left adnexa and blood This ultrasound image shows a different viewpoint of the left adnexa, including the left-sided ovary and fallopian tube. Also shown here is the presence of blood and blood flow.

**Figure 5:**
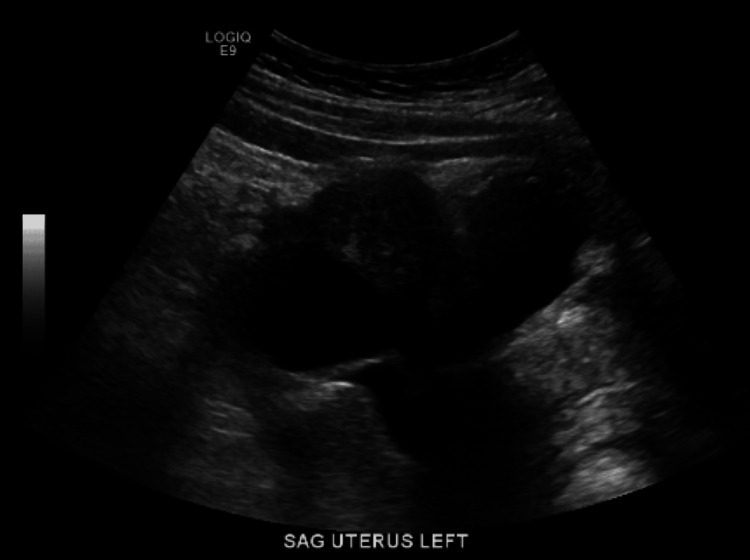
Ultrasound sagittal view of the uterus This transvaginal ultrasound (TVUS) sagittal view shows the uterus with a tiny cystic focus in the fundal endometrium. The radiology report stated that this may represent an early intrauterine gestational sac. However, pseudogestational sac and spontaneous abortion in progress or occult ectopic pregnancy could not be excluded, according to the radiology report.

Given TVUS findings and an unidentified possible ectopic pregnancy with a cystic structure of the left adnexa, a diagnostic laparoscopy along with a suction D&C was planned. Consent was obtained for diagnostic laparoscopy, suction D&C, possible salpingectomy, possible oophorectomy, and possible exploratory laparoscopy. Given the information collected from the patient’s physical exam with speculum examination, imaging, laparoscopy, and pathology results, her diagnoses were as follows: ectopic pregnancy with rupture and hemoperitoneum, right ovarian torsion with a dermoid cyst of the right ovary, subserosal fibroids, and endometriosis of the posterior cul-de-sac.

**Figure 6:**
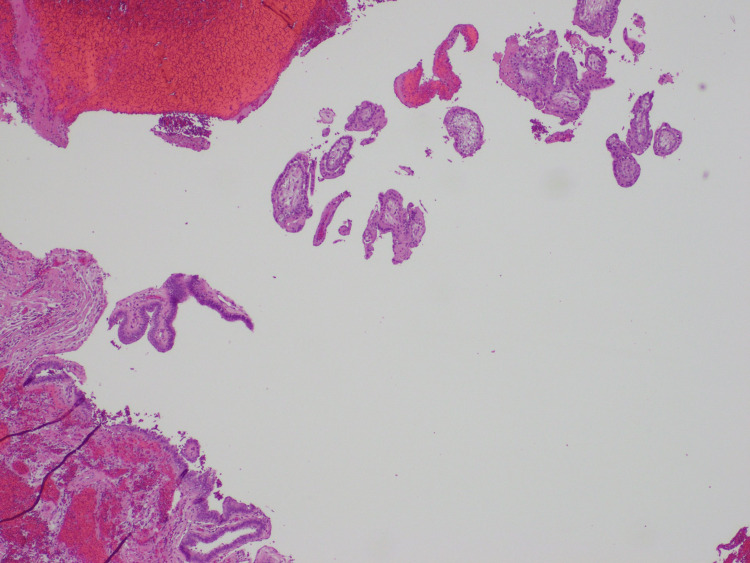
Fallopian tube epithelium This H&E stain shows the fallopian tube epithelium with chorionic villi and blood present, representing a ruptured ectopic pregnancy.

A left ectopic pregnancy was confirmed, with placental-like tissue being expelled from the fimbriated end and a hemoperitoneum of approximately 200 cc. After the left salpingectomy was performed, the specimen was removed in an Endobag and sent to pathology. The postoperative pathology report revealed fallopian tube epithelium with chorionic villi and blood present (Figure 6).

**Figure 7:**
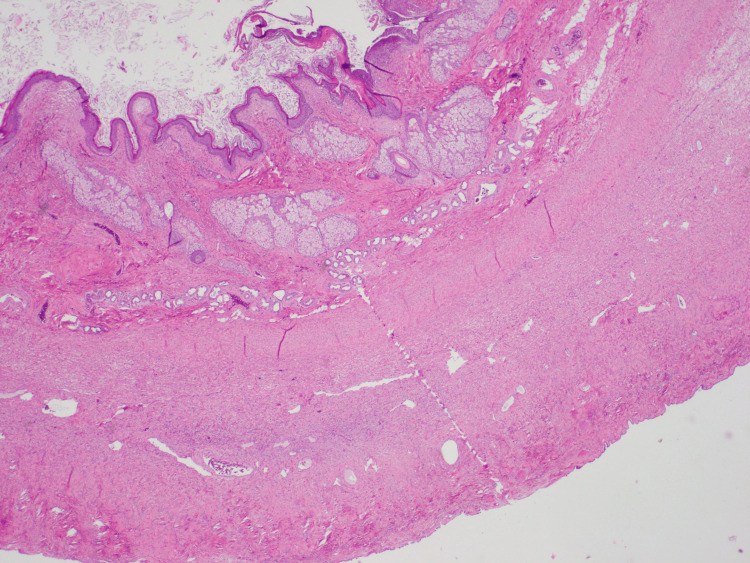
Ovarian serosal surface With this stain, the dermoid cyst is present, consisting of keratinaceous debris, squamous epithelium, sebaceous glands, hair follicles, and eccrine glands.

**Figure 8:**
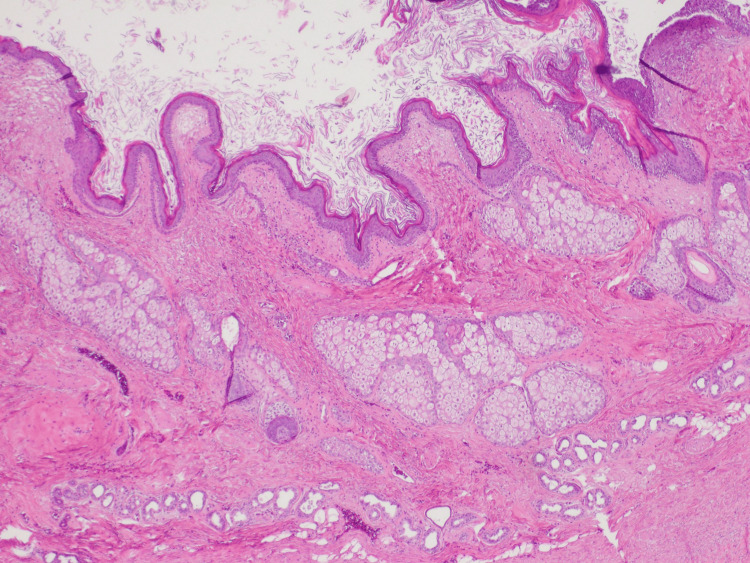
Ovarian serosal surface zoomed in Here, we provide a zoomed-in H&E stain of the ovarian serosal surface with the dermoid cyst.

A right ovarian torsion was confirmed with a right ovarian cyst of approximately 6-7 cm, which was torsed four times. A detorsion was completed prior to starting the dissection of the dermoid cyst. Upon dissection, the cyst ruptured, and fatty tissue and hair were identified. A cystectomy was performed, and the specimen was placed in an Endobag to be sent to pathology. The postoperative pathology report identified the ovarian serosal surface with the dermoid cyst consisting of keratinaceous debris, squamous epithelium, sebaceous glands, hair follicles, and eccrine glands (Figure 7-Figure 8). Copious irrigation of the pelvic cavity was completed.

Upon speculum examination under anesthesia, there was blood coming from the cervical os. On palpation of the enlarged uterus and visualization during laparoscopy, multiple subserosal uterine fibroids were noted, as well as small endometriotic implants on the posterior cul-de-sac. The patient did not follow up postoperatively or receive treatment for either her fibroids or endometriosis.

The patient was tolerating oral intake, walking on her own with pain controlled, urinating, and defecating prior to discharge on postoperative day 2. Upon discharge, vitals were within normal limits. Information for follow-up was discussed with the patient. However, the patient did not present for her postoperative follow-up visit.

## Discussion

We describe a five-week pregnant patient initially reporting pelvic pain and light vaginal bleeding who underwent a diagnostic laparoscopy, which showed evidence of a ruptured ectopic pregnancy with hemoperitoneum, right dermoid cyst with ovarian torsion, fibroids, and endometriosis. The odds of a single patient having one of these pelvic anomalies, let alone having all of them, is a rarity.

Ectopic pregnancies make up 1-2% of all pregnancies and 20% of ectopic ruptures [4]. They are characterized by similar complaints this patient presented with lateral lower quadrant pain, vaginal bleeding, nausea, and vomiting [4]. Risk factors for an ectopic pregnancy include prior ectopic, prior fallopian tube surgery/lesions/scarring, sexually transmitted disease with pelvic inflammatory disease, endometriosis, in vitro fertilization (IVF), and an intrauterine device (IUD) in place [4]. This patient had evidence of a left-sided hydrosalpinx in 2021. However, her hysterosalpingogram showed normal anatomy with patent fallopian tubes in 2022. The other known risk factor for this patient was endometriosis, which was discovered at the time of laparoscopy. According to her medical records, she had never undergone IVF and did not have an IUD in place at the time of her ectopic pregnancy.

The incidence of ovarian torsions is 2-5%; a dermoid cyst is found in 25% of all ovarian torsions [5]. Ovarian torsions can be characterized by pelvic pain or pressure, nausea, vomiting, fever, vaginal bleeding, and vaginal discharge [5]. Given the patient’s acute complaints of left lower quadrant pain and light vaginal bleeding, this was most likely occurring from the left ectopic pregnancy rather than her right-sided ovarian torsion and dermoid cyst. The risk factors for ovarian torsion include pregnancy, fertility treatments, and cysts or masses [5]. The patient was pregnant at the time of the ovarian torsion. However, the pregnancy was localized to the left fallopian tube, so the dermoid cyst most likely contributed to the ovarian torsion instead.

The estimated frequency of fibroids is 50-80% of the female population, depending on risk factors [6]. Those risk factors include lifetime changes in hormones, nulliparity, <10-year-old menarche onset, diethylstilbestrol exposure, obesity, and hypertension [6]. Fibroids may cause heavy menses, increased uterine size, infertility, pelvic pain, and constipation [6]. Given the patient’s history, the only risk factors she presented with were nulliparity and lifetime hormonal changes. She otherwise showed no signs or symptoms of fibroids, which could be the reason for the loss of follow-up for her fibroids and endometriosis.

Endometriosis affects 10-15% of females of reproductive age [7]. Risk factors include nulliparity, abnormal menstrual cycles, and first-degree family history [7]. Endometriosis is characterized by dysmenorrhea, dyspareunia, dyschezia, and infertility [7]. The patient denied dysmenorrhea, dyspareunia, dyschezia, and a family history of endometriosis. However, she was nulliparous and endorsed a history of abnormal menstrual cycles. The patient was unaware of her fibroids and endometriosis prior to the laparoscopy and never sought out treatment for these.

However, the fibroids and endometriosis were the least of her problems at the time of this evaluation. If she had not undergone laparoscopy and surgical removal, she could have developed further complications from the ectopic pregnancy and ovarian torsion, including life-threatening bleeding, ovarian necrosis, and cyst rupture, respectively. Overall, the odds of this patient presenting with all five of these anomalies simultaneously are low. However, if the laparoscopy was not performed in time, the outcome may have been substantially worse.

## Conclusions

This rare case demonstrates the possibility of multiple acute and chronic pathologies occurring in a single patient within a given time period. This case also shows that every patient experiences symptoms uniquely and may have underlying illnesses or abnormalities that have yet to make themselves known. In this particular situation, it was imperative to perform the laparoscopy given the unclear TVUS results of an unknown cystic structure concerning an ectopic pregnancy. Listening to the patient’s symptoms and acting on questionable imaging results with the next surgical step may just save the patient’s reproductive organs and/or life.
